# Harnessing natural selection to tackle the problem of prey naïveté

**DOI:** 10.1111/eva.12332

**Published:** 2015-11-17

**Authors:** Katherine E. Moseby, Daniel T. Blumstein, Mike Letnic

**Affiliations:** ^1^ School of Earth and Environmental SciencesThe University of AdelaideAdelaideSAAustralia; ^2^ Arid Recovery Ltd.Roxby DownsSAAustralia; ^3^ Department of Ecology and Evolutionary BiologyUniversity of CaliforniaLos AngelesCAUSA; ^4^ Centre for Ecosystem ScienceSchool of Biological, Earth and Environmental SciencesUniversity of New South WalesSydneyNSWAustralia

**Keywords:** exotic predators, naïveté, natural selection, predator learning, prey, reintroduction

## Abstract

Many populations are threatened or endangered because of excessive predation resulting from individuals' inability to recognize, avoid, or escape alien predators. Such prey naïveté is often attributed to the absence of prior experience and co‐evolution between native prey and introduced predators. Many reintroduction programs focus on reducing predation rate by excluding introduced predators, a focus which ignores, and indeed exacerbates, the problem of prey naïveté. We argue for a new paradigm in reintroduction biology that expands the focus from predator control to kick‐starting learning and evolutionary processes between alien predators and reintroduced prey. By exposing reintroduced prey to carefully controlled levels of alien predators, *in situ* predation could enhance reintroduction success by facilitating acquisition of learned antipredator responses and through natural selection for appropriate antipredator traits. This *in situ* predator exposure should be viewed as a long‐term process but is likely to be the most efficient and expedient way to improve prey responses and assist in broadscale recovery of threatened species**.**

## The problem of prey naïveté

Predation by introduced predators, especially mammalian predators, is a major factor responsible for the extinction of wild vertebrate populations and the failure to successfully reintroduce endangered vertebrates in many parts of the world (King 1984; Savidge 1987; Biggins et al. 1999; Johnson 2006; Moseby et al. 2011). The primary reasons for significant population declines of many native prey species and failure of subsequent reintroduction programs are thought to be (1) the inability of prey individuals to avoid and/or mount effective antipredator responses when they encounter introduced predators (Griffin et al. 2000; Short et al. 2002; Blumstein 2006; Moseby et al. 2011) and (2) the high densities and therefore high rates of encounters between introduced predators and native prey.

In many situations, introduced predators thrive in their new environments and may occur at very high population densities. There are several potential drivers of high population densities of introduced predators. These include release from constraints on population growth posed by larger predators, competitors, and parasites as well as facilitation that can occur when populations of introduced predators benefit from the presence of high densities of introduced or native prey (MacDonald and Harrington 2003; Saunders et al. 2010; Sih et al. 2010; Letnic et al. 2012). High rates of encounters between prey and over‐abundant predators can have catastrophic effects on prey populations. These population‐level effects of predators on prey are likely to be exacerbated and result in ‘hyperpredation’ on rare prey species if alternate food sources (such as over‐abundant prey) are available for predators (Sinclair et al. 1998).

While there is evidence that high densities of introduced predators can precipitate catastrophic declines in prey populations and thwart reintroduction attempts, in many instances, just one or relatively few individuals of an introduced predator species have had catastrophic impacts on populations of threatened prey (Christensen and Burrows 1994; Gibson et al. 1994; Moseby et al. 2011). These observations suggest that the susceptibility of some native prey to introduced predators is not just a function of the rate of encounters between predators and prey but also due to an asymmetry in the outcome of predator–prey encounters. For example, even when intensive feral animal control was successfully implemented at a site in inland Australia, individual exotic feral cats (*Felis catus*) were still able to cause the failure of a large‐scale mammal reintroduction program (Christensen and Burrows 1994). Similarly, Roy et al. (2002) reported that population control of an introduced mongoose (*Herpestes javanicus*) failed to prevent uncommon but significant predation events on the rare pink pigeon (*Colombus mayeri*) in Mauritius.

The high susceptibility to introduced predators displayed by many prey species is at least partly due to their naïveté (Sih et al. 2010; Carthey and Banks 2014). Naïveté can result from isolation of individuals from predators during their lifetime (ontogenetic naïveté, common in captive‐bred animals) or through an absence of co‐evolution with recently introduced predators (evolutionary naïveté, common in situations where exotic predators are present) (Griffin et al. 2000). Banks and Dickman (2007) suggested that there are three levels of naïveté to alien predators: level 1 is a failure of prey to recognize a species as a predator; level 2 is recognition of the predator but adoption of inappropriate antipredator behavior; and level 3 is where prey recognize the predator, have an appropriate response, but the predator exhibits superior hunting skills. In addition to inappropriate antipredator behavior, prey species that lack evolutionary exposure to predators may also possess other traits that make them susceptible to novel predators such as flightlessness, strong scent, noisy or conspicuous young, inadequate camouflage, and lack of nest or brood guarding behaviors.

Prey naïveté has been a particularly problematic issue for reintroduction programs in Australasia. Here, vulnerability to predation by introduced mammalian predators has caused widespread declines and extinction of more than 20 species of mammals weighing less than 5 kg in Australia (Johnson 2006) and numerous species of birds and lizards in New Zealand (King 1984; Towns et al. 2001). In some cases, populations of endangered native species remain in areas where introduced predator populations are low or absent (Johnson 2006; Innes et al. 2010). These remnant populations have been used as sources for reintroduction programs into areas of their former ranges where introduced predators exist, but in nearly all cases, reintroduction programs have failed to establish self‐sustaining wild populations (Christensen and Burrows 1994; Short and Turner 2000; Moseby et al. 2011; Hayward et al. 2012).

Predation from introduced predators has in most cases been cited as the primary cause of reintroduction failure in Australasia (Fischer and Lindenmayer 2000; Short and Turner 2000; Short 2009; Sherley et al. 2010; Moseby et al. 2011). In Australia, predation by introduced predators, the red fox (*Vulpes vulpes*), and feral cat was the reason for failure of approximately 80% of unsuccessful mammal introductions (Short 2009). In taxa such as macropodids (kangaroos, wallabies, and rat kangaroos), their susceptibility to introduced predators is so great that no safe density of introduced predators is thought to exist (Clayton et al. 2014). However, native predators can also cause reintroduction failure, particularly in captive‐bred animals, with predation and prey naivety cited as primary reasons for the failure of bird reintroductions in the Caribbean (White et al. 2005) and Saudi Arabia (van Heezik et al. 1999).

In this perspective, we propose a novel approach to overcome the problem of prey naïveté that aims to kick‐start co‐evolution between introduced predators and native prey. While much of our discussion is focused on improving the success of endangered species reintroduction programs, the concepts and approaches that we outline have broader relevance and application to ecosystems where native prey interact with introduced predators.

## Expanding the focus of reintroduction programs from predators to prey

To date, strategies to address high predation rates in reintroduction programs have largely concentrated on controlling or eradicating predators rather than improving the antipredator responses of prey (Armstrong et al. 2002; Scofield et al. 2011). However, with the exception of some island or fenced systems where a well‐defined area and the absence of immigration can facilitate predator eradication (Nogales et al. 2004), the likelihood of eradicating introduced predators is low. Consequently, many reintroduction programs focus on improving the survival of reintroduced species by reducing predator population sizes and thereby reducing the frequency of encounters between threatened prey and predators. For example, in Australia and New Zealand, one widely employed strategy to control introduced predators (primarily red foxes in Australia, and stoats (*Mustelus ermina*) and rats (*Rattus rattus*) in New Zealand) is the broad‐scale distribution of poisoned baits, often from aircraft (Burrows et al. 2003; Moseby and Hill 2011; Ruscoe et al. 2011). Another strategy that has been advocated to alleviate introduced predators impacts on prey, but remains untested in reintroduction programs, is to harness the suppressive effects that native apex predators have on populations of introduced predators (most introduced predators are mesopredators) (Crooks and Soulé 1999; Letnic et al. 2012; Hunter et al. 2015).

In larger areas, strategies for endangered species persistence that rely on suppressing or excluding populations of introduced predators may only be effective in the short term (Wayne et al. 2015). There are at least two reasons why lasting predator control is difficult to achieve. First, control techniques such as poisoning, biological control, and shooting/trapping may impose selection for predators that are less susceptible to the control technique and thus become less effective over time (Warburton and Drew 1994; Allen et al. 1996; Kohn et al. 2000). Second, programs that require perennial support from funding sources to implement predator control are vulnerable to factors beyond the control of land managers, such as variation in financial markets and the whims of funding agencies and philanthropists. Any of these reasons could lead to the failure of a reintroduction program if introduced predator populations cannot be suppressed to sufficiently low levels to allow the persistence of reintroduced species. Thus, while control of introduced predators can assist in the protection of threatened species populations (see Marlow et al. 2015), results can be short‐lived (Côté and Sutherland 1997) and there is a need to explore other alternatives for long‐term co‐existence.

In addition to evolutionary naivety, another problem facing threatened species reintroduction programs is ontogenetic naivety. Necessity and availability dictates that threatened species reintroduction programs often use captive‐bred stock or animals sourced from introduced predator‐free islands or fenced enclosures (van Heezik et al. 1999; Griffin et al. 2000; Short and Turner 2000). Indeed, in Australasia, the majority of threatened species' reintroductions now involve the transfer of individuals between predator‐free refugees (Towns and Ferreira 2001; Short 2009; Scofield et al. 2011). Although threatened prey species are kept safe from predators and reintroduction success is inevitably improved, the problem of prey naïveté is exacerbated by predator exclusion because the predator avoidance strategies of individuals from such predator‐free areas are often severely compromised (Biggins et al. 1999; McLean et al. 2000).

Complete removal of predation pressure can lead to significant and rapid loss of prey antipredator behavior through relaxed selection (Blumstein 2006; Lahti et al. 2009). This is because antipredator behaviors often involve animals increasing their antipredator vigilance at the expense of engaging in other activities, or animals avoiding certain areas because there may be an increased risk of predation. Thus, with no predators, there are no benefits from engaging in antipredator behavior and it may be rapidly lost (Blumstein et al. 2004). The often poor antipredator response of prey from predator‐free sanctuaries can hamper efforts to reintroduce individuals from these small, isolated systems into larger, natural areas in their former ranges where predators now exist.

The multipredator hypothesis (Blumstein 2006) states that prey should retain the ability to respond to predators, even extinct ones, as long as they are exposed to some predators. This is because whenever prey have more than a single predator, we expect that antipredator behaviors will not assort independently and we expect the evolution of antipredator syndromes. Thus, populations exposed to some predators, even those from different archetypes (e.g., avian but not mammalian), may retain predator discrimination abilities for all archetypes (avian and mammalian) and effective antipredator behavior even for the missing predator(s). Support for the multipredator hypothesis comes from studies that document long‐term persistence of antipredator behavior despite the loss of key predators (Byers 1997; Blumstein et al. 2004). Thus, some predation may be essential to retain the ability to respond to predators. True isolation in completely predator‐free enclosures may, however, lead to a rapid loss of antipredator abilities.

The predator archetype hypothesis predicts that if two predators are similar in some key way, species will respond to them (Cox and Lima 2006). For instance, there is remarkable morphological convergence among mammals that have similar diets and hunting styles (Wroe and Milne 2007). Additionally, related species may share similar olfactory chemicals. Support for the olfactory archetype hypothesis comes from studies of fishes trained to recognize one predator, which are more likely to generalize toward more closely related predators and less likely to generalize toward more distantly related predators (Ferrari et al. 2007). Therefore, by addressing the problem of prey naïveté rather than trying to eliminate all predators, the risks to prey from future encounters with predators may be reduced.

Another problem associated with complete removal of predators from ecosystems can arise if populations of reintroduced species increase to a point where their consumption of resources has adverse effects on the ecosystems to which they have been introduced (Hayward and Kerley 2009). Overgrazing by herbivore populations that are unchecked by natural predators has been linked to environmental degradation and fluctuations in herbivore populations in unfenced ecosystems around the world (Côté et al. 2004; Letnic et al. 2012). Overpopulation is an important consideration in predator‐free sanctuaries, because in addition to the environmental damage that can occur, there is a serious risk of catastrophic population declines of reintroduced species and adverse impacts on other species within the sanctuaries if the food resource base is exhausted (Wiseman et al. 2004; Slotow et al. 2005; Crisp and Moseby 2010; Islam et al. 2010). In some cases, supplementary feeding and/or culling is required to manage populations reintroduced into predator‐free sanctuaries after animals have over‐eaten their resource base (Hayward et al. 2007; Crisp and Moseby 2010).

These situations are clearly not natural and result in the creation of sanctuaries that are more like zoos than ecosystems (Scofield et al. 2011). While introductions to islands or fenced areas may preserve endangered species, we suggest that if ecological restoration and the establishment of self‐sustaining populations of reintroduced species is one of the aims of reintroduction programs, then we must move beyond predator‐free sanctuaries and create sustainable ecosystems. To accomplish this, we must address the inability of prey species and introduced predators to co‐exist.

Given the potential problems associated with achieving sustained control or eradication of introduced predators, we argue that there is an urgent need to expand the focus of predator management in reintroduction programs to not just focus on predator removal (which reduces the frequency of encounters between predators and prey), but to also improve the ability of prey species to avoid fatal encounters with introduced predators. While we recognize that predator detection is but one of several stages of the predation process, we suggest that if the antipredator responses of endangered prey populations could be improved, it might be more likely that introduced predators and endangered prey will co‐exist in the wild. An additional benefit of improved antipredator responses is that some tend to be broad ranging and not necessarily species specific (Blumstein 2006; Cox and Lima 2006).

## Why predator avoidance training involving simulated encounters with predators in captivity is likely not the answer

Lack of predator recognition either through evolutionary or through ontogenetic isolation is thought to be the most damaging form of prey naïveté (Cox and Lima 2006). Some reintroduction programs have attempted to improve predator recognition prior to re‐introductions by simulating encounters with predators, whereby predator cues are paired with an unpleasant experience (Griffin et al. 2000). Such prerelease predator training can modify prey behavior (Miller et al. 1990; Hölzer et al. 1995; Maloney and McLean 1995; McLean et al. 1996), and empirical evidence shows that fish (Brown and Laland 2003), birds (McLean et al. 1999), and mammals (McLean et al. 2000) can be trained to improve their antipredator skills. In some cases, trained individuals may serve as demonstrators from which other animals learn to improve their antipredator skills (Griffin and Evans 2003; Griffin 2004). If widespread, social transmission of antipredator behavior could be an effective mechanism by which animals can learn from others' experiences without direct human interventions.

Despite the promise of prerelease training coupled with natural social transmission, only a few studies have empirically tested and demonstrated that prerelease predator training can improve postrelease survival of reintroduced species (van Heezik et al. 1999; White et al. 2005). Indeed, most practitioners investigating the utility of prerelease predator training have used evidence of a prerelease change in behavior of trained individuals rather than a difference in postrelease survival of trained and untrained individuals as a measure of success (Miller et al. 1990; Hölzer et al. 1995; McLean et al. 1996; Moseby et al. 2012). Of particular note is that studies reporting improved survival of trained captive‐bred animals after release invariably involve a response to native predators rather than to exotic species (White et al. 2005; Gaudioso et al. 2011; Carthey and Banks 2014). To our knowledge, prerelease training has not been shown to reduce postrelease survival in prey species exposed to exotic predators.

In many cases, laboratory‐based predator avoidance training has been unsuccessful because captive situations do not provide the conditioning necessary for survival in the wild. Prerelease predator training often focuses on captive‐bred animals that are trained in captivity (Beck et al. 1994). Captive‐bred animals tend to have a much lower survival rate than wild animals upon release for a variety of reasons including that individuals are unfamiliar with the release site, can travel large distances, and often exhibit abnormal behavior postrelease (Snyder et al. 1996). Additionally, predator avoidance trials in captivity rarely use real predators but instead have primarily used harassment with stimuli such as with rubber bands, water pistols, stuffed animals mounted on wheels, and loud noises (McLean et al. 2000). Such unrealistic stimuli are unlikely to stimulate the fear conditioning that likely occurs naturally when an animal survives a real predatory encounter (Schakner and Blumstein 2016). Notably, one successful prerelease predator training program which did result in improved postrelease survival involved the exposure of houbara bustards (*Chlamydotis undulata*) to live predators prior to their release (van Heezik et al. 1999).

## Kick‐starting learning and natural selection

We suggest that a realistic and potentially useful form of predator avoidance training involves *in situ* predator exposure using real encounters between wild prey populations and predators. There are at least three advantages of exposing prey species to real predators. First, there may be an increased capacity for learning and reinforcement. Second, there is the opportunity to select for individuals with improved antipredator behaviors through natural selection. Third, predators not humans will do the selecting and thus avoid biases toward particular traits that can be readily observed or quantified by humans.

Learning is improved because the stimuli are real, the exposed population is wild, and cultural transmission can occur during all life stages. Several studies have reported improved learning of antipredator behavior in captive situations when actual live predators are used rather than predator models or scent (White et al. 2005; Carthey and Banks 2014). Additionally, appropriate predator avoidance behavior is likely to be strongly reinforced during *in situ* predator exposure due to prolonged exposure to the predator and the opportunities for filial and cultural transfer (Griffin et al. 2000).

Perhaps more importantly, *in situ* predator exposure allows us to harness the effects of natural selection to select for appropriate traits. We envisage that selection by predators is unlikely to operate on a single trait, but on a suite of characters that could conceivably include behavioral, physiological, and physical traits. Thus, we propose that strong selection pressure imposed by introduced predators on some naïve prey species should improve their capacity to avoid fatal encounters with predators. However, it is important to note that the traits linked to improved survival which predators select for may not be readily observable or quantifiable by humans except as improved survival. Thus, an advantage of using *in situ* predator exposure may be that it could reduce biases toward selection for readily measurable traits imposed by humans and instead select for the traits that demonstrably confer greater longevity and reproductive success.

In the case of behavioral responses to predators, the relative significance of learning versus natural selection in improving antipredator behavior is likely to be influenced by the sociality of the prey species. Solitary species may rely more on natural selection and filial transfer, while social species may have improved opportunities for learning from conspecifics (Griffin et al. 2000). We caution, however, that learning or enhancing innate antipredator responses to native predators lost through ontogenetic isolation is likely to be substantially easier than developing new, effective antipredator responses against introduced predators due to evolutionary naïveté.

As evidenced by the extinction of predator‐exposed populations of naïve prey, a major obstacle to overcome when using *in situ* predator exposure would be the magnitude of the selection pressure that predators can impose on naïve prey. A solution may be to expose prey to predators under tightly controlled conditions, where the rate of prey mortality can be closely monitored, and the predators removed should there be a risk that the prey population could become extinct.

From a viewpoint focused on maintaining genetic diversity, it may appear risky to select threatened species for a suite of traits associated with antipredator responses, because such selection could reduce the population's genetic diversity. However, this initial loss of genetic diversity is likely to be offset if improved learning and the long‐term co‐existence of prey species with exotic predators eventually lead to larger populations of threatened species and, ultimately, greater genetic variation. We suggest that if prey populations are to survive with exotic predators in the long term, then the genetic bottlenecking that inevitably results from a bout of natural selection will be desirable if it improves threatened species' capacity to co‐exist with introduced predators. Preserving genetic diversity in captive breeding programs requires significant time and effort. Most diversity is lost when animals are released and high mortality from predation occurs. In the case where prey naïveté and predation from exotic predators are the most important factors causing population extinction, we consider selecting for traits associated with enhanced survival when confronted with introduced predators to be both justifiable and essential: some reduction in genetic variation is better than the complete failure to recover a population in the wild.

Are there populations of threatened prey species large enough to conduct *in situ* predator exposure? Predator‐free islands and fenced sanctuaries offer the best opportunities to manage this *in situ* process. In some cases, these areas experience an oversupply of threatened prey species due, ironically, to an absence of natural predators. Sterilized and radio‐collared single predators could be added to islands and fenced enclosures where healthy populations of threatened prey are present while leaving adjacent areas untouched as insurance populations. We assume that predators will explore this environment and leave signs of their presence (e.g., scent marks, vocalizations) that prey can learn to associate and potentially avoid. We assume that some prey will be attacked and some may be killed. Importantly, we assume that some prey will directly survive encounters with predators, while others might have the opportunity to learn vicariously through others' experiences. These are realistic assumptions. Many prey species identify and respond to the scents and sounds of their predators, even novel predators that resemble natural predators (Kohn et al. 2000; Blumstein et al. 2002; Blumstein 2006; Anson and Dickman 2013; Gérard et al. 2014). Previously, naïve survivors have been shown to modify their behavior after direct or vicarious experiences with predators (Berger et al. 2001). Additionally, previous single predator incursions have been recorded in fenced reserves and have not resulted in mass predation events, suggesting that many prey species can tolerate the low densities of predators required for these trials (see examples in Moseby et al. 2015).

Given these assumptions, prey that survive will have either learned to avoid predation, or undergone a bout of selection through which survivors passed on whatever heritable traits that facilitated survival to the next generation. There are many examples of environmental change associated with human activities and biological invasions imposing strong selection pressures on species and in doing so driving rapid phenotypic change (Phillips and Shine 2004; Darimont et al. 2009). Such changes can both be evolutionary and/or represent phenoptypic plasticity (Kohn et al. 2000; Hendry et al. 2008). The capacity of native species to respond to *in situ* training is likely to vary considerably depending on vulnerability of each life stage to predation and the opportunities that exist for filial and cultural transfer. We suggest that it is essential to understand the life‐history strategies a species uses as well as documenting baseline naiveté to identify potential responsive populations and species for such *in situ* training.

How do we predict if and when natural selection by predators will sufficiently reduce prey vulnerability in existing predator environments? This can be tested through both intergenerational comparisons and experimental manipulations. The changes in intergenerational prey responses can be measured by quantifying how prey respond to predator cues using scents, sounds, and models as well as how their vigilance and escape behavior (e.g., flight initiation distance) vary (Cooper et al. 2015). Additionally, the survival rates of successive generations can be tested during *in situ* predation experiments: increased survival demonstrates improved antipredator responses.

When differences are detected, manipulations can be conducted to determine when prey are ‘ready’ to face environments that support predator populations. These can be conducted by gradually adding more *in situ* predators up to the densities recorded in existing predator environments and measuring the responses of prey. Ultimately, successive generations of *in situ* trained prey would be released into existing predator areas with varying predator densities and their survival compared with untrained individuals. Understanding the predation thresholds that can be tolerated by untrained and progressively trained prey will also assist with understanding the limitations of *in situ* predator training and the importance of simultaneous predator control.

An important consideration for programs that use *in situ* predation to improve reintroduced species antipredator responses will be the threshold level of population reduction at which the experiment is ceased. Theory predicts that the stronger the selection (i.e., the more animals killed), the more likely the resulting population will be different from the original population in its antipredator behavior. Massive reductions of a threatened or endangered species, however, may not be practical or desirable. In practice, the threshold level of population reduction using *in situ* predation will be determined by trade‐offs between factors such as the number of individuals of the reintroduced species population, the expected rates of intrinsic growth and natural mortality, and the capacity to remove the predator. Such demographic factors could be modeled prior to initiating an experiment.

## Ethical considerations

Is this *in situ* predator exposure ethically defensible? Will it result in excessive mortality of endangered species? What about the welfare of individual prey species and predators?

We adopt here an explicitly ecologistic perspective (e.g., Kellert 1976; Simaika and Samways 2010) because our primary goal as conservation biologists and ecologists is to help recover populations and restore ecosystems. By contrast, Vucetich and Nelson (2007) emphasize the importance of thinking about the welfare of individuals that may suffer in a conservation intervention. While our focus on population recovery does not mean that we should not be concerned with the welfare of individuals, it does focus our goals on the ultimate goal of recovering populations of threatened or endangered species. We believe that wildlife managers need more effective tools and approaches to recover populations, particularly those where failure is in response to predation on recently released animals.

We believe that the lessons from reintroduction biology have shown us that captive‐bred animals have a particularly high mortality rate upon release into the wild and that this is often driven by predation. In cases where naïve prey encounter introduced predators, predation may be absolute. Thus, some have argued that reintroductions, given their low success rate, are ethically questionable (Bekoff 2002). However, increasing populations of endangered species and creating sustainable wild populations in some jurisdictions are mandated by laws such as the Endangered Species Act of the USA. If we aim to restore ecosystems where introduced predators interact with naïve prey, something bold must be performed. Viewed this way, there is a moral imperative to explore novel management strategies.

Does this need to develop more effective introduction strategies that outweigh the costs to individual prey and predators that might be involved in such an intervention? We believe it could, and therefore, it is certainly worth exploring the idea with a set of well‐designed experiments. For our proposed interventions to work, some individual prey will likely die. While we understand that not all will agree with our viewpoint, we believe that if *in situ* predation improves the antipredator responses of endangered prey, and populations are ultimately recovered, the individual suffering imposed on individual prey which might be killed while in an enclosure with a captive predator is outweighed by the benefits of recovering the population by programs which aim to reduce the rate of encounters between predators and prey by killing predators.

Issues relevant to the welfare of the predator are worthy of discussion and debate. We envision two types of predators that would be used in this sort of experiment: evolutionarily novel ones and native predators. Neither directly benefits from *in situ* predator exposure; indeed, they are used as tools for its implementation.

Consider introduced cats and foxes in Australia. Introduced by Europeans, these species have had a disproportionately negative impact on small and mid‐sized Australian mammals (Johnson 2006). Thus, in many places, these introduced predators are primarily poisoned and to a lesser degree trapped and shot to reduce population density or eliminate them in fenced reserves (Moseby et al. 2011). Nonetheless, native Australian mammals must interact with them if they are to survive outside a fenced reserve. Thus, it may be defensible to use these abundant predators, who otherwise would be killed, in experiments to determine the efficacy of *in situ* predator exposure if their welfare is taken into account. Furthermore, if *in situ* predation is successful at improving the antiresponses of endangered mammals, it may negate the need to kill introduced predators in the future and so reduce the suffering experienced by predators. By contrast, consider a native predator and a native prey. Depending on the situation, it might warrant further discussion and debate about whether to use a native canid, dingoes in the Australian example, as a tool to prepare a small mammal for release. We think that these are questions that must be discussed and debated on a case‐by‐case basis (e.g., Vucetich and Nelson 2007).

The ultimate goal of *in situ* predator exposure is to reduce mortality rates so that the rate of predator‐driven mortality does not exceed the rate of population growth. Recent studies have called for consideration of both the ecological and evolutionary cost of resource management decisions (Ashley et al. 2003), emphasizing that evolutionary changes can occur over relatively short time frames (Thompson 1998; Hendry et al. 2008). For instance, managers already recognize that genetic changes can occur over short time frames in animals subjected to captive housing as they adapt to captive conditions (Williams and Hoffman 2009). Recommendations for improving reintroduction success using captive‐bred animals include minimizing the number of generations in captivity (Williams and Hoffman 2009). Closer integration of evolutionary biology with reintroduction biology is likely to improve environmental outcomes through what Carroll et al. (2014 pg. 1) term ‘manipulating the relationships between the traits of organisms and the patterns of selection imposed by their environments’. Our suggestion of using *in situ* predation to force rapid behavioral and potentially genetic change is one example of how these manipulations and integrations could be used to improve reintroduction success. We do not envision this technique being possible to adopt in all cases where we have threatened populations of captive animals, but in those cases where it is possible, we think that it may ultimately be a useful tool that might even result in a reduced need to kill extant predators.

## A paradigm shift

To date, there has been little focus on the role of prey naïveté in the decline of threatened wildlife species despite introduced predators being a prominent cause of species declines. Introductions of the red fox to California and subsequent fauna declines (Lewis et al. 1999) show that the impacts of introduced predators are not confined to island ecosystems or specific countries. The global risk of future predator incursions is high, fueled by the deliberate and inadvertent movements of species by humans. Reviews have highlighted the importance of advancing our understanding of prey naïveté (Carthey and Banks 2014) particularly in relation to introduced predators. We call for novel strategies to help wildlife biologists protect and re‐establish populations of endangered wildlife and strongly encourage a shift in focus from exclusion of introduced predators to improving prey responses in order to facilitate future resilience and possible co‐existence. This paradigm shift from predator exclusion to co‐existence would not diminish the importance of predator control, but rather assist in facilitating an integrated, multilevel approach to threatened species management.

The effectiveness of *in situ* predator exposure as a method of improving the antipredator traits of wildlife prey species should be evaluated. First, we must know whether animals have learned or the traits of populations have changed after being exposed to a predator. This can be evaluated by comparing the antipredator behavior, physical and physiological traits, and survival of predator‐exposed versus predator naïve individuals. Second, we must know whether these experiences with predators actually increase reintroduction success outside of predator‐free sanctuaries. Detailed tracking of control versus predator‐experienced animals and their progeny will be essential to determine the ultimate success of this strategy.

With proper monitoring, *in situ* predator exposure can be ethically defensible. Ultimately, *in situ* predator exposure has the potential to revolutionize reintroduction biology and significantly change the way that future faunal reintroductions are designed and implemented. Indeed, if the goal is to restore ecosystems that contain both predators and their prey, such techniques may be essential.
